# Health and economic impacts of China’s rural Toilet Revolution

**DOI:** 10.2471/BLT.25.295599

**Published:** 2026-08-20

**Authors:** Zhenyu Zhang, Qijiong Zhu, Runming Du, Jiarui Wang, Zhisheng Liang, Jingtao Ding, Xiangqun Zheng, Yujun Shen, Zhijie Zheng

**Affiliations:** a Academy of Agricultural Planning and Engineering, Ministry of Agriculture and Rural Affairs, Beijing, China.; b Department of Global Health, Peking University School of Public Health, 38 Xue Yuan Road, Haidian District, Beijing 100191, China.; c Peking University Third Hospital, Beijing, China.; d Institute of Environment and Sustainable Development in Agriculture, Chinese Academy of Agricultural Sciences, Beijing, China.

## Abstract

**Objective:**

In 2015, China implemented the national Toilet Revolution, which aimed to upgrade toilets in rural areas. We quantified the health and economic impact of the Toilet Revolution from 2018 to 2024 by examining the association between sanitary toilet coverage and the incidence of diseases transmitted by the faecal–oral route.

**Method:**

We used national and provincial data on sanitary toilet coverage and the reported incidence of faecal–oral diseases. We adjusted estimated associations by socioeconomic and environmental covariates. We calculated population-attributable fractions to estimate the preventable disease burden, and assessed economic benefits through reductions in treatment costs associated with a reduction in cases of faecal–oral diseases.

**Findings:**

From 2018 to 2024, the estimated rural sanitary toilet coverage increased from an estimated 56% to 76%, while faecal–oral disease incidence declined substantially. A 1% increase in toilet coverage was associated with a 1.56% reduction in national incidence of disease. Overall, 16.87% (95% confidence intervals: 16.64 to 17.12) of the observed decline in the incidence of faecal–oral diseases was attributable to increased toilet coverage. This reduction corresponded to average annual treatment savings of 4.93 million yuan (¥; 1 United States dollar ≈ ¥ 6.80) and cumulative savings exceeding ¥ 200 million. Forward projections indicate that continued expansion of sanitary toilet coverage will further reduce disease incidence up to 2032.

**Conclusion:**

Our analysis shows that provision of sanitation infrastructure is a high-impact population health intervention that produces sustained economic returns at scale.

## Résumé

**Objectif:**

En 2015, la Chine a lancé la Révolution des toilettes, initiative visant à moderniser les installations sanitaires dans les zones rurales. Nous avons quantifié les impacts sanitaires et économiques de la Révolution des toilettes entre 2018 et 2024 en examinant le lien entre la disponibilité des toilettes sanitaires et l’incidence des maladies transmises par voie fécale-orale.

**Méthodes:**

Nous avons employé des données nationales et provinciales sur la disponibilité des toilettes sanitaires et l’incidence déclarée des maladies transmissibles par voie fécale-orale. Nous avons ajusté l’estimation des associations en fonction de covariables socio-économiques et environnementales. Nous avons calculé les fractions attribuables à la population afin d’estimer la charge de morbidité évitable et évalué les avantages économiques grâce à la réduction des coûts de traitement liée à la diminution des cas de maladies transmissibles par voie fécale-orale.

**Résultats:**

De 2018 à 2024, la disponibilité estimée des toilettes sanitaires en milieu rural est passée de 56% à 76%, tandis que l’incidence des maladies transmissibles par voie fécale-orale a considérablement diminué. Une augmentation de 1% du taux de disponibilité des toilettes était associée à une réduction de 1,56% de l’incidence nationale de ces maladies. En tout, 16,87% (intervalles de confiance à 95%: 16,64 à 17,12) de la baisse observée de l’incidence des maladies transmissibles par voie fécale-orale étaient imputables à l’augmentation du taux de disponibilité des toilettes. Cette réduction correspondait à une économie annuelle moyenne de 4,93 millions de yuans (¥; 1 dollar américain ≈ 6,80 ¥) en frais de traitement et à une économie cumulée supérieure à 200 millions ¥. Les projections indiquent qu’une disponibilité encore plus grande de toilettes sanitaires permettra de réduire davantage l’incidence des maladies jusqu’en 2032.

**Conclusion:**

Notre analyse montre que la mise en place d’infrastructures sanitaires constitue une intervention de santé publique à fort impact qui génère des retombées économiques durables à grande échelle.

## Resumen

**Objetivo:**

En el año 2015, China puso en marcha la “Revolución de los Inodoros”, cuyo objetivo era mejorar el estado de las letrinas en las zonas rurales del país. Cuantificamos el impacto sanitario y económico de la “Revolución de los Inodoros” de 2018 a 2024 analizando la asociación entre la cobertura de letrinas higiénicas y la incidencia de enfermedades transmitidas por la vía fecal-oral.

**Métodos:**

Utilizamos datos nacionales y provinciales sobre la cobertura de letrinas higiénicas y la incidencia notificada de enfermedades de transmisión fecal-oral. Ajustamos las asociaciones estimadas en función de covariables socioeconómicas y ambientales. Calculamos las fracciones atribuibles poblacionales para estimar la carga de enfermedad prevenible y evaluamos los beneficios económicos mediante la reducción de los costes de tratamiento asociados a una disminución de los casos de enfermedades de transmisión por vía fecal-oral.

**Resultados:**

Entre 2018 y 2024, la cobertura estimada de letrinas higiénicas en las zonas rurales aumentó de un 56% a un 76% aproximadamente, mientras que la incidencia de las enfermedades de transmisión fecal-oral disminuyó de forma significativa. Un aumento del 1% en la cobertura de letrinas se asoció con una reducción del 1,56% en la incidencia nacional de la enfermedad. En general, el 16,87% (intervalos de confianza del 95%: de 16,64 a 17,12) del descenso observado en la incidencia de enfermedades de transmisión fecal-oral se debió al aumento de la cobertura de inodoros. Esta reducción correspondió a un ahorro promedio anual en el tratamiento de 4,93 millones de yuanes (¥; 1 dólar estadounidense ≈ 6,80 ¥) y un ahorro acumulado superior a 200 millones ¥. Las proyecciones a futuro indican que la expansión continuada de la cobertura de letrinas higiénicas reducirá aún más la incidencia de enfermedades hasta el año 2032.

**Conclusión:**

Nuestro análisis muestra que la provisión de infraestructura de saneamiento es una intervención de salud pública de alto impacto que genera rendimientos económicos sostenidos a gran escala.

## ملخص

**الغرض:**

في عام 2015، قامت الصين بتنفيذ ثورة المراحيض الوطنية التي هدفت إلى الارتقاء بالمراحيض في المناطق الريفية. قمنا بقياس الأثر الصحي والاقتصادي لهذه الثورة في الفترة من 2018 إلى 2024 من خلال دراسة العلاقة بين معدلات تغطية المراحيض الصحية، ومعدلات الإصابة بالأمراض التي تنتقل عبر مسار البراز والفم.

**الطريقة:**

وقمنا باستخدام بيانات وطنية وإقليمية حول تغطية المراحيض الصحية ومعدلات الإصابة المُبلغ عنها بالأمراض المنقولة عبر مسار البراز والفم. كما قمنا بتعديل العلاقات التقديرية حسب المتغيرات المصاحبة الاجتماعية والاقتصادية والبيئية. وحسبنا النسب المنسوبة للسكان لتقدير عبء المرض الذي يمكن الوقاية منه، وقيّمنا الفوائد الاقتصادية من خلال التخفيضات في تكاليف العلاج المرتبطة بانخفاض حالات الإصابة بالأمراض المنقولة عبر مسار البراز والفم.

**النتائج:**

ففي الفترة ما بين 2018 و2024، ارتفعت نسبة تغطية المراحيض الصحية في المناطق الريفية من %56 إلى %76 بشكل تقديري، بينما انخفضت معدلات الإصابة بالأمراض المنقولة عبر مسار البراز والفم بشكل ملحوظ. وارتبطت الزيادة بنسبة %1 في تغطية المراحيض بانخفاض قدره %1.56 في معدل الإصابة الوطني بالمرض. وبشكل عام، كان ما نسبته %16.87 (بفاصل ثقة %95: 16.64 إلى 17.12) من الانخفاض الملحوظ في معدلات الإصابة بالأمراض المنقولة عبر مسار البراز والفم يعود إلى زيادة تغطية المراحيض. وقد أدى هذا الانخفاض إلى توفير سنوي في تكاليف العلاج بمتوسط 4.93 مليون يوان (حيث يعادل الدولار الأمريكي الواحد حوالي 6.80 يوان)، وإلى توفيرات تراكمية تجاوزت 200 مليون يوان. وتشير التوقعات المستقبلية إلى أن استمرار التوسع في تغطية المراحيض الصحية سيؤدي إلى مزيد من الانخفاض في معدلات الإصابة بالأمراض حتى عام 2032.

**الاستنتاج:**

ويُظهر تحليلنا أن توفير البنية التحتية للصرف الصحي يُعد تدخلاً صحياً سكانياً عالي الأثر، ويحقق عوائد اقتصادية مستدامة على نطاق واسع.

## 摘要

**目的:**

中国于 2015 年实施了全国性厕所革命，其目标是升级改造农村地区的厕所。通过研究卫生厕所覆盖率与粪口传播疾病发病率之间的关联，我们对 2018 年至 2024 年间厕所革命的健康和经济影响进行了量化评估。我们利用了全国性和省级层面关于卫生厕所覆盖率及上报粪口传播疾病发病率的数据。

**方法:**

我们利用社会经济和环境协变量对估算的关联进行了调整。我们计算了人群归因分数，以估算可预防疾病负担，同时评估了与粪口传播疾病病例数减少相关的治疗费用下降所带来的经济效益。

**结果:**

据估算，2018 年至 2024 年间，农村卫生厕所覆盖率从大约 56% 增至 76%；而粪口传播疾病的发病率则大幅降低。卫生厕所覆盖率每扩大 1%，对应全国粪口传播疾病发病率下降 1.56%。总体来说，在观察到的粪口传播疾病发病率下降中，有 16.87%（95% 置信区间：16.64 至 17.12）可归因于厕所覆盖率的扩大。这相当于年均节省治疗费用 493 万元人民币（1 美元约等于 6.8 元人民币），累计节省金额超过了 2 亿元人民币。前瞻性预测表明，直至 2032 年，继续扩大卫生厕所覆盖率都将进一步降低粪口传播疾病发病率。

**结论:**

我们的分析显示，提供卫生基础设施是高影响力的人群健康干预措施，能够大规模产生持续的经济回报。

## Резюме

**Цель:**

В 2015 году в Китае началась общенациональная «туалетная революция», целью которой была модернизация туалетов в сельской местности. Была проведена количественная оценка влияния «туалетной революции» на здоровье населения и экономику в период с 2018 по 2024 год посредством изучения связи между охватом территории санитарно-гигиеническими туалетами и уровнем заболеваемости болезнями, передающимися фекально-оральным путем.

**Методы:**

Были использованы общенациональные данные и данные по провинциям об охвате санитарно-гигиеническими туалетами и зарегистрированной заболеваемости фекально-оральными инфекциями. Полученные взаимосвязи были скорректированы с учетом социально-экономических факторов и факторов окружающей среды. Для оценки предотвратимого бремени болезней были рассчитаны популяционные атрибутивные доли, а экономическая выгода оценивалась по снижению затрат на лечение, обусловленному сокращением числа случаев фекально-оральных заболеваний.

**Результаты:**

В период с 2018 по 2024 год расчетный охват санитарно-гигиеническими туалетами в сельской местности увеличился примерно с 56% до 76%, в то время как заболеваемость фекально-оральными инфекциями существенно снизилась. С ростом охвата туалетами на 1% отмечалось снижение общенационального показателя заболеваемости на 1,56%. В целом 16,87% (95%-й ДИ: от 16,64 до 17,12) наблюдаемого снижения заболеваемости фекально-оральными инфекциями было обусловлено увеличением охвата туалетами. Данное снижение привело к среднегодовой экономии на лечении в размере 4,93 млн юаней (1 доллар США ≈ 6,80 юаня), а совокупная экономия превысила 200 млн юаней. Прогнозы на будущее показывают, что дальнейшее расширение охвата санитарно-гигиеническими туалетами обеспечит дополнительное снижение заболеваемости вплоть до 2032 года.

**Вывод:**

Проведенный анализ демонстрирует, что обеспечение санитарно-технической инфраструктуры является высокоэффективным средством в сфере охраны здоровья населения, которое приносит устойчивый экономический эффект в масштабах всей страны.

## Introduction

Safe sanitation is a cornerstone of public health. Inadequate sanitation, together with unsafe water and poor hygiene, contributes to diarrhoeal disease, neglected tropical infections and environmental contamination. Globally, despite two decades of progress, substantial gaps remain. In 2022, an estimated 3.5 billion people still lacked safely managed sanitation, including about 419 million who practised open defecation and, in 2024, more than 3.4 billion people were still without safely managed sanitation services at home.^1^ In China, between 2000 and 2020, access to sanitary toilets (toilets that meet basic hygiene and sanitation standards) in mainland China increased from an aggregated estimate of 19% to 78%, with particularly rapid gains in rural areas.^2^ These improvements build on decades of state-led public health campaigns but since 2015 they have accelerated because of the national so-called Toilet Revolution, which made the upgrading of toilets in rural areas a flagship of rural revitalization and environmental governance. However, coverage and service levels still vary across regions and socioeconomic groups, and enteric infections are still a sizeable burden. For example, a recent analysis estimated that about 75 million incident cases of enteric infection and more than 5500 deaths occurred in China in 2021, despite significant declines in age-standardized rates since 1990.^3^

Evidence on the health effects of sanitation improvements increasingly suggests that sanitary toilets provide important but context-dependent benefits.^4^ Global randomized trials and meta-analyses indicate that sanitation and broader water, sanitation and hygiene interventions can reduce diarrhoea by roughly 15–25% on average, but effect sizes vary widely by setting, baseline coverage and co-interventions.^5^^,^^6^ Indeed, some recent large trials reported small or null effects when sanitation was improved in isolation.^5^ In China, microlevel studies using national survey data have found that household access to sanitary toilets is associated with better self-rated health and lower recent diarrhoea in rural adults, after accounting for socioeconomic factors.^7^ Longitudinal household survey data from rural China further suggest that higher village-level sanitary toilet coverage may benefit the wider community.^8^ Ecological analyses from some provinces and national surveillance data similarly report that increases in sanitary toilet coverage coincide with declines in intestinal infectious diseases.^9^

Despite this growing body of evidence, important gaps remain. Most Chinese studies focus on single provinces or pre-2015 programmes, rely on cross-sectional designs or self-reported morbidity, and typically evaluate toilets as individual household exposures rather than as an infrastructure system whose health effects depend on coverage, quality, and sustained operation and maintenance. To address these gaps, we aimed to: (i) quantify the association between provincial-level sanitary toilet coverage and the incidence of the main diseases transmitted by the faecal–oral route during the Toilet Revolution period; (ii) project the investment needed to expand sanitary toilets; and (iii) distil China’s policy, financing and technology pathways into a transferable framework for low- and middle-income countries.

## Data sources

### Funds for the Toilet Revolution

China’s Toilet Revolution has been supported by different funding systems over the past 2 decades. The central government has allocated substantial financial resources through direct subsidies, transfer payments and performance-based incentives.^10^ We obtained data on the central funds supporting the Toilet Revolution in China from 2019 to 2024 from the Ministry of Agriculture and Rural Affairs. Although some provinces did not receive central funds in certain years due to policy adjustments, the data collected over the whole study period nonetheless covered all 31 provinces, autonomous regions and municipalities in mainland China.

We obtained national- and province-level data on sanitary toilet coverage from the Ministry of Agriculture and Rural Affairs (online repository).^11^ Sanitary toilet coverage was defined as the proportion of rural households using toilets that meet national standards for rural sanitary toilets, including the safe containment of human excreta to prevent human contact. These systems include water-flush toilets connected to sewerage or treatment systems, three-compartment septic tanks, biogas-digester toilets, double-pit alternating toilets and other locally approved sanitary systems.

### Incidence of faecal−oral diseases

We restricted outcome data to four nationally notifiable faecal–oral diseases with consistent national and province-level annual data from 2018 to 2024: cholera, dysentery, hepatitis A, and typhoid and paratyphoid fever. More common enteric pathogens, including diarrhoeagenic *Escherichia coli* and norovirus, are monitored through outbreak investigations, foodborne-disease surveillance, laboratory testing networks and environmental or sentinel monitoring. However, these pathogen-specific surveillance streams did not provide consistently published incidence series by province and year for the full study period. We therefore did not include these pathogens as outcome variables in the main analysis. We calculated the incidence rates based on the resident population and the number of disease cases in China for 2022 and 2024 (online repository).^11^

### Covariates

We collected data on the daily per capita domestic water consumption, local expenditure on medical and health care, local expenditure on environmental protection, and resident population by province and year. We extracted these data from the China Statistical Yearbooks published by the National Bureau of Statistics of China.

## Analysis

We calculated average annual percentage changes of the incidence of faecal−oral diseases and the sanitary toilet coverage rate using linear regression models. We used generalized linear mixed models with a Poisson distribution to examine the association between the sanitary toilet coverage rate and the incidence of faecal−oral diseases. The model incorporated covariates, including daily per capita domestic water consumption, local expenditure on medical and health care, and local spending on environmental protection. We incorporated a random intercept for province to account for clustering effects across provinces. We included the natural logarithm of the province-specific population size in the generalized linear mixed models as an offset term. Health behaviour changes (e.g. handwashing and toilet use) may jointly drive sanitation benefits, but we could not quantify these effects because the relevant data were not available. Instead, we conducted a sensitivity analysis with additional adjustment for education level and health education delivery to test the robustness of the results, as these metrics partly reflect the extent of health behaviour changes (online repository).^11^ We also estimated the potential health and economic benefits from the Toilet Revolution (online repository).^11^ Detailed information on the study design and methods is provided in the online repository.^11^

## Developing a framework

We developed a structured framework to synthesize implementation of rural sanitation. We reviewed relevant national and local policy documents to identify institutional arrangements and implementation responsibilities. We classified financing mechanisms across the construction and operation phases, and developed a decision tree for selecting suitable toilet and excreta-treatment systems based on local environmental conditions, available infrastructure, whether households are clustered or dispersed, and user preferences. We also reviewed records of international cooperation, particularly the Ministry of Agriculture and Rural Affairs–United Nations Children’s Fund (UNICEF) collaboration, to identify external inputs related to capacity-building, technical support pilot demonstrations and knowledge exchange.

## Results

### Toilet coverage and disease incidence

From 2018 to 2024, the estimated aggregated national coverage of sanitary toilets in rural China increased from 56.00% to 76.00%, with an average annual percentage change of 5.25% (95% confidence intervals, CI: 3.25 to 7.29). Over the same period, annual central government funds earmarked for the Toilet Revolution remained close to 7 billion Chinese yuan (¥; 1 United States dollar (US$) ≈ ¥ 6.80) in most years (Table 1). Between 2018 and 2024, the incidence of dysentery declined from 6.57 to 2.47 cases per 100 000 population (average annual percentage change −16.35%, 95% CI: −21.07 to −11.35) and typhoid and paratyphoid fever from 0.78 to 0.34 cases per 100 000 (average annual percentage change −12.51%, 95% CI: −15.40 to −9.52). Hepatitis A showed a modest non-significant downward trend from 1.17 to 1.02 cases per 100 000 (average annual percentage change −6.10%, 95% CI: −13.76 to 2.24). Cholera incidence remained low throughout (≤ 0.002 cases per 100 000 annually) and showed no clear time trend (average annual percentage change −2.98%, 95% CI: −30.63 to 35.69). Fig. 1 shows the trend in faecal–oral disease incidence nationally by the increase in sanitary toilet coverage from 2018 to 2024. At the provincial level in 2018, toilet coverage was lower in western and northern China compared with eastern and southern regions, but it increased rapidly between 2018 and 2024 (Table 2).

**Table 1:** Toilet Revolution funding, coverage of sanitary toilets and incidence of diseases transmitted by the faecal−oral route, China, 2018−2024

Parameter	Year							Average annual % change (95% CI)
	2018	2019	2020	2021	2022	2023	2024	
**Central funds (billion ¥)^a^**	NA	7.00	7.38	4.82	7.40	7.40	6.87	NA
**Estimated coverage of sanitary toilets, %**	56	60	68	70	73	75	76	5.25 (3.25 to 7.29)
**Incidence, no. of cases per 100 000 population**								
Cholera	0.002	0.001	0.0008	0.0004	0.002	0.002	0.0007	−2.98 (−30.63 to 35.69)
Dysentery	6.57	5.81	4.12	3.58	2.55	2.63	2.47	−16.35 (−21.07 to −11.35)
Hepatitis A	1.17	1.38	1.05	0.85	0.77	0.82	1.02	−6.10 (−13.76 to 2.24)
Typhoid and paratyphoid	0.78	0.66	0.50	0.51	0.41	0.39	0.34	−12.51 (−15.40 to −9.52)

CI: confidence intervals; NA: not applicable; ¥: yuan.

^a^ 1 United States dollar ≈ ¥ 6.80.

**Fig. 1:**
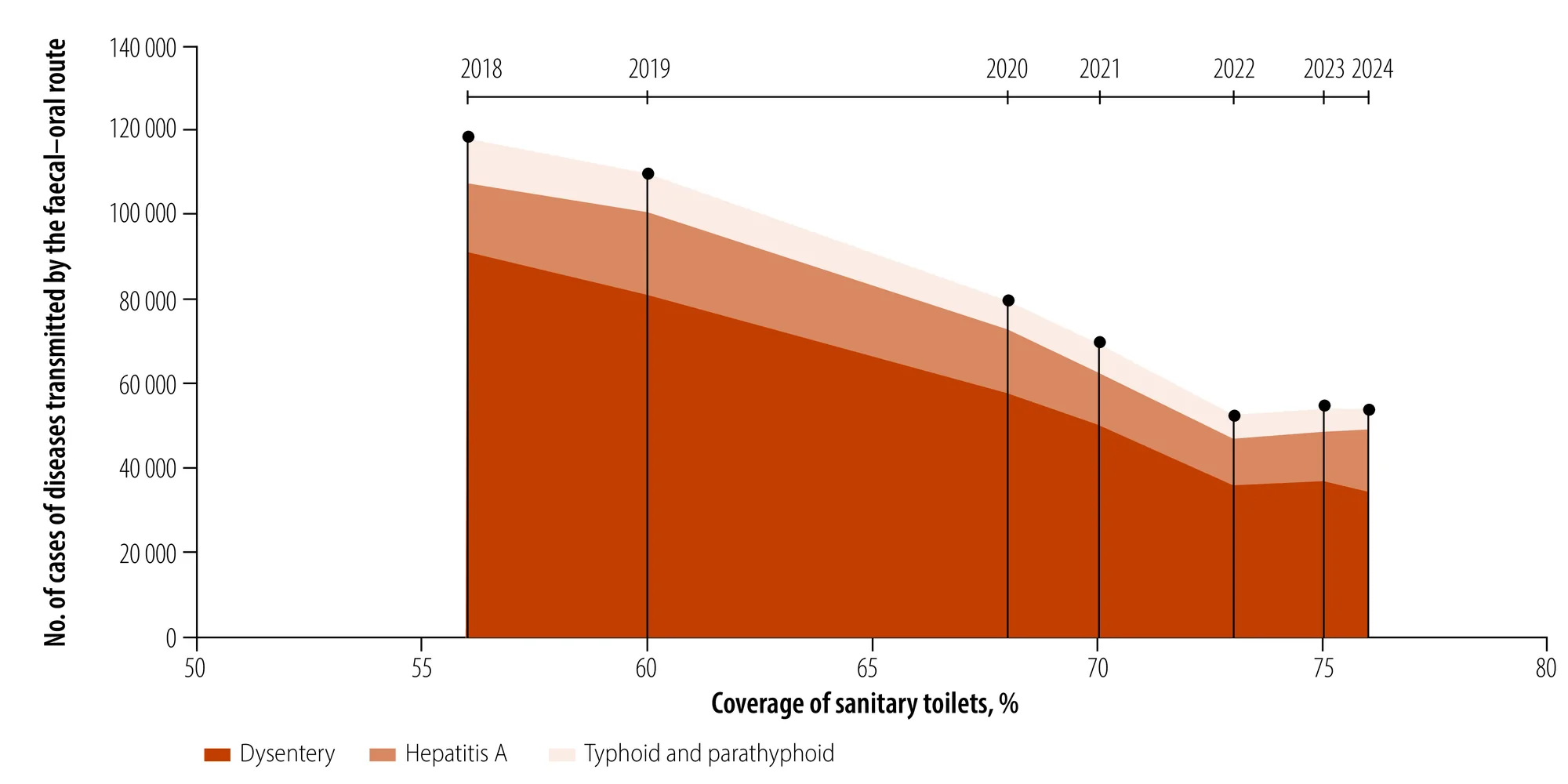
Incidence of diseases transmitted by the faecal−oral route and coverage of sanitary toilets during the Toilet Revolution, China, 2018−2024

**Table 2:** Sanitary toilet coverage, by province, China, 2018-2024

Province or area	Reported sanitary toilet coverage, %		Change in coverage, %
	2018	2024	
Beijing	89.5	99.0	9.5
Tianjin	86.0	98.0	12.0
Hebei	36.4	81.1	44.7
Shanxi	25.0	78.0	53.0
Inner Mongolia	22.4	56.8	34.4
Liaoning	79.2	83.0	3.8
Jilin	NA	67.2	NA
Heilongjiang	80.1	85.0	4.9
Shanghai	NA	100.0	NA
Zhejiang	99.6	100.0	0.4
Anhui	80.1	90.0	9.9
Fujian	97.6	100.0	2.4
Jiangxi	88.9	94.0	5.1
Shandong	92.3	92.3	0.0
Henan	79.0	85.0	6.0
Hubei	75.1	93.0	17.9
Hunan	75.3	93.0	17.7
Guangdong	96.7	97.0	0.3
Guangxi	86.4	95.3	8.9
Hainan	92.2	99.5	7.3
Chongqing	72.6	87.8	15.2
Sichuan	59.8	95.0	35.2
Guizhou	75.0	78.0	3.0
Yunnan	21.9	81.9	60.0
Tibet	25.4	80.3	54.9
Shaanxi	46.0	83.1	37.1
Gansu	12.9	82.0	69.1
Qinghai	31.9	67.3	35.4
Ningxia	31.9	69.0	37.1
Xinjiang	28.0	84.9	56.9
Jiangsu	97.6	99.0	1.4
Hong Kong SAR	NA	NA	NA
Macao SAR	NA	NA	NA

NA: not available; SAR: special administrative region.

Across provinces and years, higher sanitary toilet coverage was strongly associated with lower incidence of the four major faecal–oral diseases. Each percentage increase in sanitary toilet coverage was associated with an estimated 1.56% (95% CI: 1.54 to 1.58) reduction in the national incidence of these diseases. Results from our sensitivity analysis indicated the robustness of our findings (online repository).^11^ Using reducible population-attributable fractions and setting 2018 as the baseline coverage level, we estimated that 16.87% (95% CI: 16.64 to 17.12) of the observed decline in faecal–oral disease incidence between 2018 and 2024 was attributable to increases in sanitary toilet coverage during the Toilet Revolution period.

Fig. 2 shows the estimated additional treatment costs for faecal–oral diseases attributable to insufficient sanitary toilet coverage between 2018 and 2024. Over this period, the decline in faecal–oral disease incidence was accompanied by a gradual reduction in the corresponding treatment costs. Nationally, the reduction in disease incidence from 2018 to 2024 was associated with a significant average annual decrease of ¥ 4.93 million in treatment expenditures (*P*-value: 0.002), with cumulative savings of more than ¥ 200 million over the study period.

**Fig. 2:**
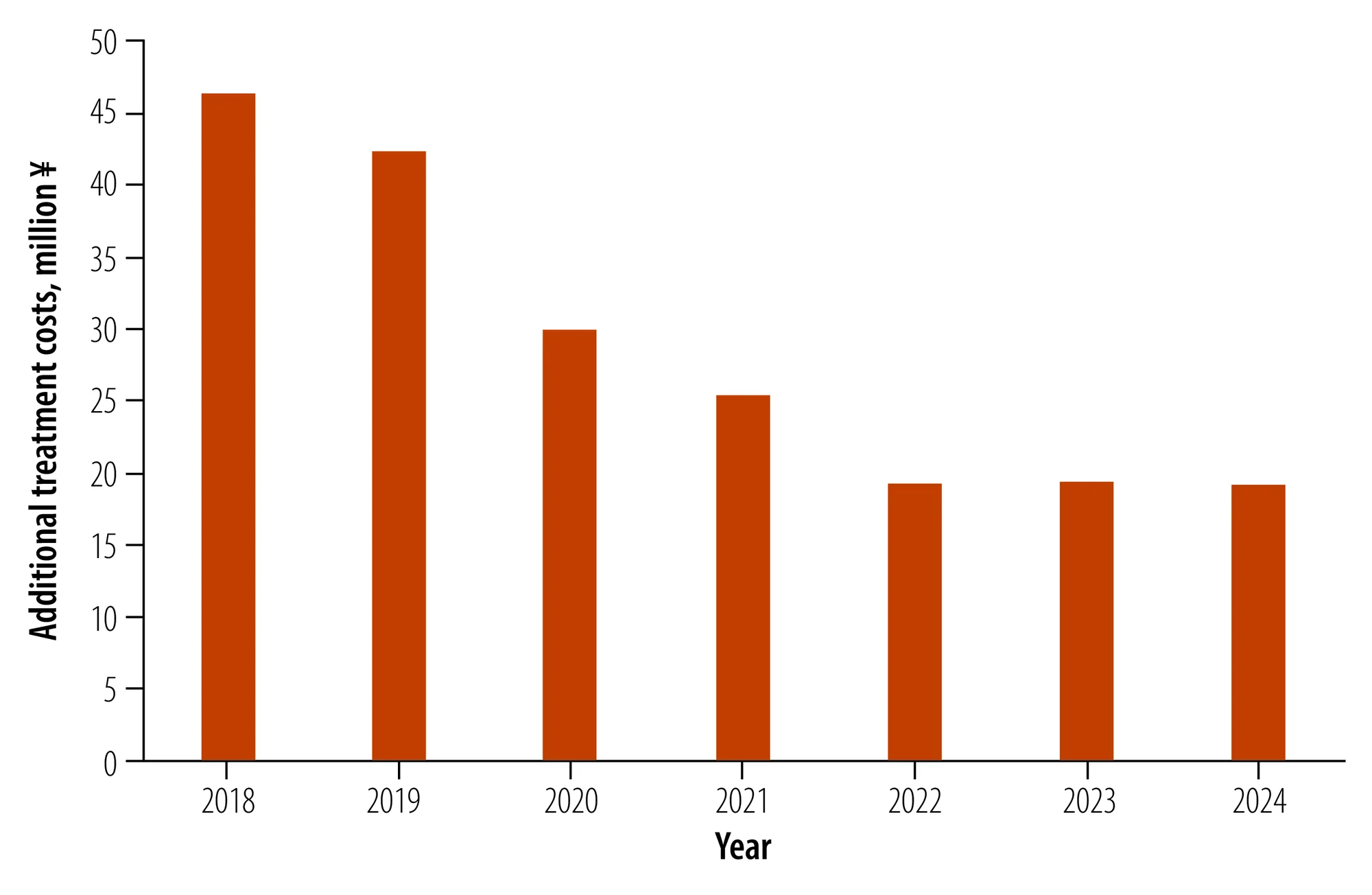
Estimated additional treatment costs for diseases transmitted by the faecal−oral route attributable to insufficient toilet coverage, China, 2018−2024

¥: yuan.

1 United States dollar ≈ ¥ 6.80.

### Funding to expand toilet coverage

Fig. 3 shows the observed and projected relative risks of faecal–oral diseases and the corresponding central government investments in the Toilet Revolution for the period 2018–2032. For 2018–2024, the curves show the historical trajectory of risk and annual central investment. For 2024–2032, the figure gives projections of sanitary toilet coverage, associated risks of faecal–oral diseases and required annual investments based on the observed relationship between investment and coverage growth. The projected curves indicate that in the 5 years after 2024, the annual investment will remain at a relatively high level and the relative risk of faecal–oral diseases will continue to decline. After 2029, as provincial coverage approaches the planned completion levels, the projected central investment requirement will decrease substantially, while the projected relative risk curve gradually flattens in the later years of the projection period.

**Fig. 3:**
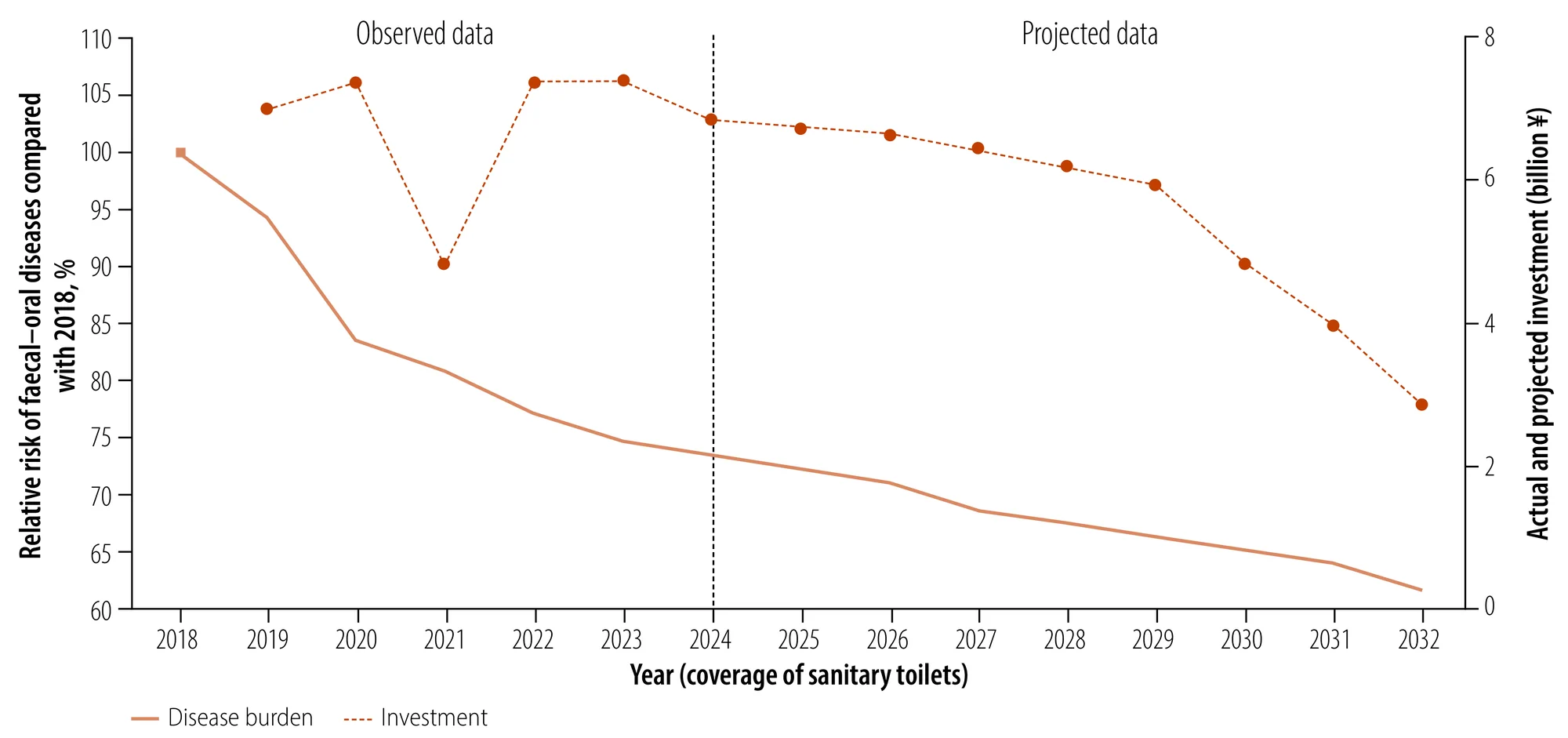
Projected risk of diseases transmitted by the faecal−oral route and required investments, China, 2018−2032

¥: yuan.

1 United States dollar ≈ ¥ 6.80.

### Transferable framework

Our study identified an integrated implementation framework linking policy, funding and technology selection. Policy provided the regulatory basis by defining strategic priorities, institutional responsibilities, standards and implementation requirements. Financing arrangements differed by phase. Initial construction was supported by central-government funding, matching funds from provincial, municipal and county governments, and household contributions. Operation and maintenance, which has received greater attention only in recent years, relied mainly on subnational subsidies, market-based financing and service provision by social enterprises or third-party operators, and household payments rather than routine central-government funding. Technology selection was guided by local environmental and infrastructure conditions, settlement patterns, user preferences, the intended reuse of treated excreta and financial feasibility. Because technologies differ in construction and recurrent costs, available financing shaped which options could be adopted and sustained, while the selected technology determined subsequent operation and maintenance requirements (Fig. 4). We identified international inputs as a supporting component rather than a main driver; in particular, UNICEF contributed through capacity-building activities, technical training, pilot demonstrations, support with technology selection and knowledge exchange.

**Fig. 4:**
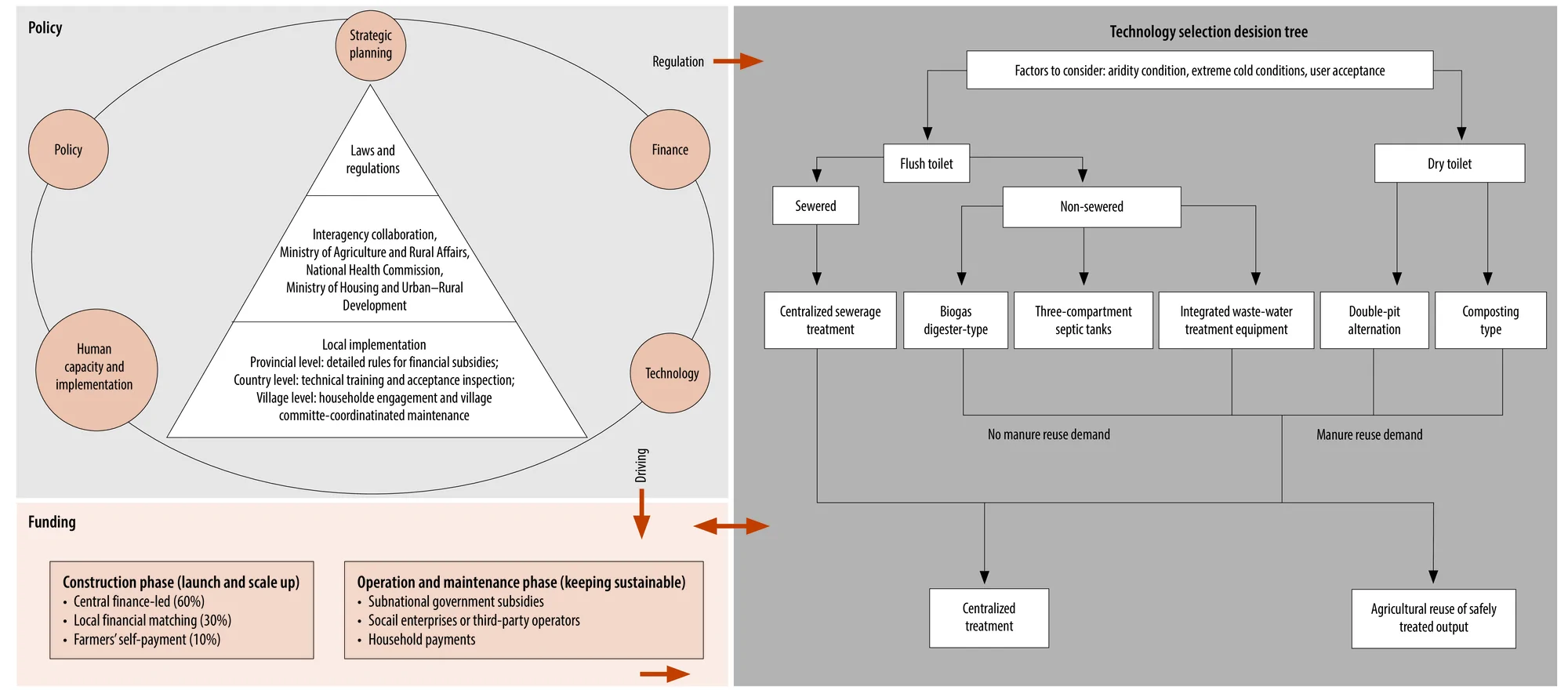
Adaptable rural sanitation framework based on China’s Toilet Revolution for low- and middle-income countries

Notes: we organized the framework around four pillars: policy, finance, technology, and human capacity and implementation. In the policy section the optional enhancements can be scaled up or down according to financial resources and implementation capacity. In the funding section, central government funding refers to funds allocated by the central government at the national level, whereas subnational government subsidies refer to subsidies provided by subnational governments at the provincial, municipal or county level. The approximate financing shares shown for the construction and scale-up phase were informed by consultations with relevant government authorities. They are indicative rather than nationally standardized proportions and vary across localities.

## Discussion

In this nationwide analysis covering the first decade of China’s rural Toilet Revolution, we found that rapid expansion of sanitary toilet coverage was accompanied by marked declines in the incidence of four main faecal–oral diseases. These decreases in incidence were associated with measurable savings in national treatment expenditure.

Our results are broadly consistent with, and extend, previous evidence on the impact of sanitation on health. Meta-analyses of sanitation and broader water, sanitation and hygiene interventions have reported relatively small average reductions in diarrhoeal disease, with substantial heterogeneity across settings and interventions.^5^ Large trials have suggested that household-level toilet improvements alone may have a limited effect in highly contaminated environments or where coverage is low.^12^^,^^13^ In China, survey-based studies have linked household access to sanitary toilets with better self-rated health and lower self-reported diarrhoea among rural adults.^7^^,^^14^ Additionally, provincial^9^ and county^15^ studies have shown inverse associations between sanitary toilet coverage and intestinal infectious diseases. Against this background, our study adds national, policy-relevant evidence from the recent Toilet Revolution period, showing that increases in sanitary toilet coverage have measurably contributed to declines in faecal–oral infections and to reductions in direct medical expenditure at the population level.^3^ Taken together, this body of work suggests that the key question is no longer whether sanitation matters for health, but how sanitation systems need to be designed and implemented to maximize and sustain these benefits.

In China, our findings need to be interpreted in the context of rural sanitation implementation. The Toilet Revolution has relied predominantly on improved on-site systems, especially three-compartment septic tanks, often combined with subsequent treatment, discharge or reuse of effluent and sludge.^2^^,^^16^ The performance of these systems varies by climate and geographic conditions: cold climates, high groundwater tables, karst terrain and water-scarce regions all pose challenges for effective containment and the subsequent safe treatment of faecal sludge and effluent. In many rural areas, domestic sewage treatment has lagged behind toilet construction, and where flush toilets have been rapidly promoted, the additional hydraulic and pollutant load on underdeveloped wastewater infrastructure can create new environmental pressures.^17^ These technical and environmental constraints indicate that the next phase of China’s Toilet Revolution must focus on aligning toilet technologies with local hydrogeologic conditions, water availability and treatment capacity, and on shifting from simple coverage targets to safely managed sanitation across the whole service chain.

Financing, operation and maintenance, and user behaviour are important challenges for consolidating and extending the gains documented in our paper. Central government allocations for the Toilet Revolution have averaged at around ¥ 7 billion a year in recent years, but financial capacity and prioritization vary widely across provinces and counties. Capital subsidies have facilitated the large-scale construction and upgrading of rural toilets, yet recurrent costs for desludging, maintenance and repair are often underfunded or responsibility for them falls ambiguously between households, village committees and local governments. This responsibility differs by facility type: household toilets are primarily maintained by users, village-level communal toilets are generally cleaned and maintained by cleaners hired or arranged by village committees, and school or workplace toilets are managed outside the rural Toilet Revolution framework through their respective institutional operators. In some communities, newly built sanitary toilets are underused or modified because of persistent preferences for traditional practices, concerns about smell or reliability, or limited understanding of the links between sanitation and health. These issues suggest that future phases of the Toilet Revolution will need to move from a construction-oriented approach to one that places greater emphasis on sustainable financing of operation and maintenance, clear delineation of responsibilities along the service chain, and sustained engagement with rural households and communities to support behaviour change and consistent toilet use.

International comparisons suggest that China’s Toilet Revolution should be seen as a systems-oriented reference model rather than a prescriptive blueprint. India’s Swachh Bharat Mission–Grameen illustrates a mission-mode pathway centred on household incentives, mass behaviour change mobilization, digital monitoring and village-level implementation. On the other hand, Indonesia’s sanitation reform follows a more decentralized and community-led pathway, with greater emphasis on facilitation, local planning, partner support and gradual system strengthening (Table 3). Compared with these approaches, China’s model places emphasis on top-level political prioritization, dedicated public financing, integration with broader rural development agendas and context-specific technology selection. These differences indicate that the transferable value of China’s experience lies less on replicating its exact financing scale or administrative arrangements, and more on its system logic, that is, aligning policy commitment, predictable funding, clear operation-and-maintenance responsibilities, behavioural support, routine monitoring and locally appropriate technology choices. For low- and middle-income countries with more resource constraints, core functions such as political prioritization, partial public financing for both construction and recurrent costs, explicit cost-sharing rules, and basic monitoring and behaviour-change mechanisms may be minimally required. The exact subsidy level, central–local–household cost split and market-based resource-recovery models should be adapted to national income, fiscal capacity and private-sector readiness. In this sense, China, India and Indonesia offer complementary lessons: China provides a systems-planning template, India a mobilization-and-monitoring template and Indonesia an adaptation-and-local-ownership template for advancing safely managed rural sanitation.

**Table 3:** Comparison of national rural sanitation programmes, China, India and Indonesia

Feature	China	India	Indonesia
Programme and period	Toilet Revolution, launched in 2015; rural sanitation scale-up accelerated from 2017 and later linked to rural revitalization	Swachh Bharat Mission–Grameen, launched in 2014: phase I focused on ending open defecation, phase II on sustaining gains and managing waste	National rural sanitation reform built around community-based total sanitation, expanded from the mid-2000s through water and sanitation programmes
Governance model	Strong central political leadership, with local governments responsible for implementation, and rural sanitation embedded in broader rural governance and development agendas	Centrally sponsored national mission, implemented through a clear administrative chain from national to village levels	More decentralized and multiagency model, with health, public works, planning agencies and local governments sharing responsibilities
Financing structure	Mainly public financing, combining central support, local matching of funds and household contributions, with subsidy levels varying by locality rather than based on one national formula	Much of the capital cost covered by central and state governments with household incentives used to promote toilet construction; funding for waste management and sustainability added in phase II	Relied on national and local government budgets, donor-supported programmes and community-based grants, with funding focused on facilitation and local capacity rather than universal household hardware subsidies
Implementation instruments	Administrative mobilization, financial support, local adaptation, technical guidance and integration with rural development priorities	Large-scale behaviour-change campaign, toilet construction incentives, digital monitoring and village-level mobilization	Community-led behaviour change, local planning, training, facilitation, and partner support for monitoring and implementation
Overall orientation	A state-led systems approach linking policy, funding, technology choice and local implementation	A mission-mode campaign combining mass mobilization, household incentives and monitoring	A decentralized, community-based approach emphasizing behaviour change, local ownership and gradual system strengthening

Notes: we obtained information from national policy documents and programme reports. Key sources include: China: Three-Year Action Plan for Improving the Rural Living Environment (2018–2020)^10^ and related policy documents on the Rural Toilet Revolution issued by the General Office of the Communist Party of China Central Committee and the State Council. India: Swachh Bharat Mission–Grameen guidelines.^18^^,^^19^ Indonesia: Community-Based Total Sanitation Strategy and related water and sanitation programme documents, including PAMSIMAS.^20^^,^^21^ Additional information was supplemented by peer-reviewed literature and international programme reports.

Our study has several strengths. First, we used recent, nationally representative routine data to link trends in sanitary toilet coverage with surveillance-based incidence of important faecal–oral diseases during a period of intense policy focus on rural sanitation. Second, by combining disease incidence with attributable fractions and unit treatment costs, our analysis goes beyond relative risks to quantify the contribution of sanitation to changes in both health burden and health-care expenditure. Finally, we integrated empirical trends with forward-looking projections of investment needs and disease risks, providing a policy-relevant view of the returns of sustained investment in rural sanitation.

Our study also has some limitations. First, the analysis is ecological and conducted at the provincial level, so causal inferences at the individual or community level cannot be drawn. Unmeasured confounding by other time-varying factors, such as improvements in drinking-water quality, hygiene promotion, poverty alleviation, health-care access, surveillance practices, health behaviours and cognitive changes, may partially account for the observed trends. Second, coverage measures were based on administrative statistics and may not fully capture toilet functionality, safe containment or actual use. Our estimates, therefore, reflect the impact of sanitary toilet coverage as reported, rather than the broader concept of safely managed sanitation. Third, we focused on four notifiable faecal–oral diseases and excluded other outcomes that may be affected by sanitation, such as non-notifiable enteric infections, environmental enteric dysfunction or certain neglected tropical diseases. Fourth, the cost analysis was restricted to direct medical expenditure and used average treatment costs that may vary across settings and over time. As such, our estimates likely understate the full economic benefits of reduced disease, including productivity gains and avoided household out-of-pocket spending. Finally, projections of future disease risks and investment requirements were based on historical relationships and simplifying assumptions and should be interpreted as illustrative scenarios rather than precise forecasts.

In conclusion, expansion of sanitary toilet coverage in rural China was associated with marked reductions in faecal–oral disease incidence and lower national-level treatment expenditure. These quantitative estimates show that large-scale, publicly supported sanitation programmes can yield measurable health and economic gains.
